# Association Between Workday Sleep Deprivation, Weekend Catch-Up Sleep, and Abdominal Adiposity Indicators: A Cross-Sectional Study Among Brazilian Female Fixed-Shift Workers

**DOI:** 10.3390/diseases14020043

**Published:** 2026-01-28

**Authors:** Anderson Garcez, Sofia Vilela, Janaína Cristina da Silva, Ingrid Stähler Kohl, Harrison Canabarro de Arruda, Maria Teresa Anselmo Olinto

**Affiliations:** 1 Programa de Pós-Graduação em Ciências Médicas: Endocrinologia, Universidade Federal do Rio Grande do Sul (UFRGS), Porto Alegre 90610-264, Brazil; ingridkohl.nutri@gmail.com; 2 EPIUnit/ITR, Instituto de Saúde Pública da Universidade do Porto, Universidade do Porto (UP), 4050-600 Porto, Portugal; sofia.vilela@ispup.up.pt; 3 Programa de Pós-Graduação em Saúde Coletiva, Universidade do Vale do Rio dos Sinos (Unisinos), São Leopoldo 93022-000, Brazil; cds.janaina@hotmail.com; 4 Programa de Pós-Graduação em Alimentação, Nutrição e Saúde, Universidade Federal do Rio Grande do Sul (UFRGS), Porto Alegre 90610-264, Brazil; harrisoncanabarro@gmail.com

**Keywords:** sleep, sleep deprivation, abdominal fat, shift work, women

## Abstract

Background: Sleep deprivation may contribute to increased abdominal adiposity. Although weekend catch-up sleep is associated with various health outcomes, its role in abdominal adiposity remains unclear, particularly among female fixed-shift workers. Therefore, this study aimed to explore the association of workday sleep deprivation and weekend catch-up sleep with abdominal adiposity indicators in Brazilian female fixed-shift workers. Methods: A cross-sectional study was conducted on 450 female fixed-shift workers aged ≥ 18 years from a large industrial group in Southern Brazil. Abdominal adiposity indicators linked to cardiovascular risk were assessed: waist circumference (WC ≥ 88 cm), waist-to-height ratio (WHtR > 0.5), weight-to-waist index (WWI ≥ 11), conicity index (C-Index ≥ 1.27), and WC & Body Mass Index (combined WC ≥ 88 cm and BMI ≥ 30 kg/m^2^). Workday sleep deprivation was defined as <6 h (h) of sleep on workdays, and weekend catch-up sleep (absolute difference between weekend and workday sleep duration) was defined as >2 h longer sleep on weekends vs. workdays. Associations were estimated using a Poisson regression with robust variance adjusted for demographic, socioeconomic, behavioral, reproductive, and occupational confounders. Results: The mean age was 34.9 ± 9.9 years. The prevalence rates of abdominal adiposity were 45.3% for WC, 47.6% for WHtR, 26.2% for WWI and C-Index, and 28.7% for WC&BMI. Workday sleep deprivation and weekend catch-up sleep were reported by 27.1% and 43.3% of the participants, respectively. After adjustment for confounders, workday sleep deprivation was consistently associated with higher abdominal adiposity: Prevalence Ratio (PR) = 1.37 (95% CI: 1.10–1.69) for WC; 1.25 (95% CI: 1.02–1.53) for WHtR; 1.48 (95% CI: 1.07–2.04) for WWI; 1.43 (95% CI: 1.03–1.99) for C-Index, and 1.59 (95% CI: 1.17–2.16) for WC&BMI. Longer weekend catch-up sleep was positively associated with WHtR (PR = 1.24; 95% CI: 1.03–1.49) and WC&BMI (PR = 1.39; 95% CI: 1.04–1.85). Conclusions: Workday sleep deprivation was consistently linked to increased abdominal adiposity, whereas associations with longer weekend catch-up sleep were less consistent. These findings underscore the potential metabolic risk of insufficient sleep among female shift workers.

## 1. Introduction

Abdominal adiposity, characterized by excessive accumulation of visceral fat in the abdominal region, is a major public health concern because of its strong association with increased cardiovascular risk and mortality [1,2,3]. Evidence suggests that abdominal adiposity indicators, such as waist circumference, are more reliable predictors of cardiovascular disease and mortality than general measures of obesity, such as Body Mass Index (BMI) [4,5,6]. The global prevalence of central obesity is estimated at approximately 41.5%, with a significant increase observed over the past three decades [7]. Notably, higher prevalence rates have been reported among specific subgroups, particularly women, and regionally, the highest rates have been documented in South America, reaching 55.1% [7]. These findings highlight the critical importance of assessing abdominal fat distribution when evaluating cardiovascular risk.

Sleep-related factors, including sleep deprivation, which is defined as insufficient sleep for environmental or personal reasons [8], have been linked to a range of adverse health outcomes, including cardiovascular diseases and increased mortality [9,10]. Furthermore, sleep deprivation may contribute significantly to the development of abdominal adiposity. Evidence from a recent systematic review and meta-analysis of prospective cohort studies consistently identified short sleep duration (<6 h per day) as a significant risk factor for central obesity in adults [11].

Habitual sleep deprivation during workdays is often unavoidable due to the demands of modern lifestyles and work/school schedules. As a result, many people cope by extending their sleep on non-workdays to compensate—a pattern commonly referred to as ‘catch-up sleep’ or ‘weekend catch-up sleep’ [12,13,14]. Previous studies have explored the potential relationship between weekend catch-up sleep and various health outcomes [12,13,14]; however, contradictory findings have been reported. For instance, a weekend catch-up sleep duration exceeding 2 h was associated with a lower prevalence of cardiovascular disease among sleep-deprived participants in the 2017–2018 National Health and Nutrition Examination Survey (NHANES) [15]. In contrast, a weekend catch-up sleep duration of at least 2 h was not associated with mortality or the incidence of cardiovascular disease in a population-based prospective cohort study conducted in the United Kingdom [16].

To date, only one study has specifically examined the association between weekend catch-up sleep and abdominal adiposity, showing that individuals with weekend catch-up sleep had an 18% higher risk of abdominal obesity compared with those with sufficient sleep, based on data from Korean workers participating in the 2016–2023 Korean National Health and Nutrition Examination Survey (KNHANES) [17]. Nevertheless, independent of weekly sleep duration, previous studies reported inconsistent findings regarding the relationship between weekend catch-up sleep and obesity. A cross-sectional study in the Republic of Korea found that each additional hour of weekend catch-up sleep was associated with a 0.12 kg/m^2^ reduction in BMI [18]. Similarly, results from the seventh Korea National Health and Nutrition Examination Survey (KNHANES VII) showed a significant negative association with BMI in individuals with ≤0 h of catch-up sleep, while no such association was observed in those with >0–1, >1–2, or >2 h [19]. Conversely, the SONAR-Brazil Survey reported that >2 h of weekend catch-up sleep was linked to a 78% higher risk of overweight and a 1.61 kg/m^2^ increase in BMI [20].

Shift work, which usually involves irregular or unusual hours, such as night work and/or rotating shifts, is increasingly adopted in modern society as a mechanism to enhance flexibility and productivity in work schedule organization compared to conventional daytime work [21]. However, shift work is often associated with sleep deprivation, primarily due to disruptions in the circadian rhythm and sleep–wake cycle, with sleep duration being significantly influenced by variations in shift start and end times [22,23]. Additionally, previous studies have reported a higher prevalence of abdominal adiposity [24,25], and permanent night workers have been shown to have a 29% higher risk than rotating shift workers [26].

Therefore, this study aimed to explore the association of workday sleep deprivation and weekend catch-up sleep with abdominal adiposity indicators in Brazilian female fixed-shift workers. This addresses an important gap in the literature, as no previous research has investigated these associations in this population, which might be particularly vulnerable to sleep restriction due to domestic and occupational factors.

## 2. Materials and Methods

### 2.1. Study Design and Population

This cross-sectional study included a sample of female fixed-shift workers from factories of a large industrial group involved in manufacturing household plastic products in the metropolitan region of Porto Alegre, Rio Grande do Sul, Brazil. We analyzed the data collected in 2022/2023 as part of the project ‘Health Conditions of Shift-Working Women: Longitudinal Occupational Health Study (ELO Saúde),” which was approved by the Ethics Committee of the University of Vale do Rio dos Sinos (approval no. 5681627). All participants provided written informed consent, and all procedures complied with ethical standards, including informed consent, confidentiality, and anonymity, in accordance with the Declaration of Helsinki on research involving human subjects.

### 2.2. Sample

**Figure 1:**
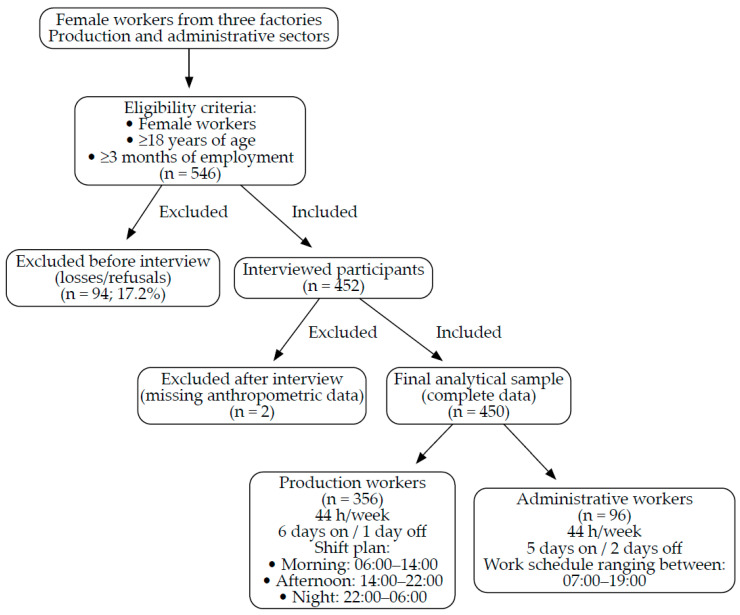
Flowchart of the study sample and work schedules/shift plan.

The sample included female workers from three factories encompassing both the production and administrative sectors. All female workers aged 18 years or older with at least three months of employment in the industrial group were considered eligible for inclusion. The exclusion criteria were pregnancy or absence from work at the time of data collection. Of the 546 eligible female workers, 452 were interviewed after accounting for loss or refusal (17.2%). Subsequently, two were excluded because of missing anthropometric data, resulting in a final sample of 450 individuals with complete data. The final sample comprised 356 production and 96 administrative workers, all working 44 h per week. Production workers had a six-day schedule with one day off, whereas administrative workers had a five-day schedule with two days off. The participant selection process, inclusion and exclusion criteria, and work schedule characteristics are summarized in Figure 1.

### 2.3. Data Collection and Instruments

Data were collected from August 2022 to March 2023 using a standardized, precoded, and pretested questionnaire developed for this study, which included both self-reported items and direct anthropometric measurements. Data were collected through in-person interviews conducted with trained professionals at designated workplaces. A pilot study was previously conducted to test the instruments and train interviewers. To assess data quality, 10% of the interviews were randomly re-administered by telephone using a simplified questionnaire focused on items with low short-term variability (e.g., education level and usual sleep schedule, including bedtime and wake time). No discrepancies were found, indicating the high reliability of the data collection.

Objective anthropometric measurements included body weight, height, and waist circumference (WC). Body weight (kg) was measured using a calibrated digital anthropometric scale (Omron^®^ model HN-289LA, OMRON Healthcare, São Paulo, Brazil) with a maximum capacity of 150 kg and a precision of 100 g. Body height (m) was measured using a portable vertical stadiometer (Balmak^®^ model EST-223, BALMAK, São Paulo, Brazil) with a measurement range from 0 to 2.1 m and a precision of 1 mm [27]. Waist circumference (cm) was measured using a non-extendable tape measure with 1 mm accuracy. Waist circumference was measured directly on the skin at the midpoint between the last rib and the top of the iliac crest, with participants standing in a relaxed expiratory position [28]. All measurements were standardized and performed in duplicate, with the participants in an orthostatic position, wearing light indoor clothing, having emptied their pockets, and no shoes. The average of the two measurements was used to derive indicators of abdominal adiposity.

Research supervisors performed data coding, and the collected data were double-entered using the EpiData software version 3.1 (Centers for Disease Control and Prevention, Atlanta, GA, USA) and subsequently checked for typographical and entry errors.

### 2.4. Outcomes: Abdominal Adiposity Indicators

In this study, we calculated and analyzed multiple indicators of abdominal adiposity as outcome variables based on established cutoff points linked to elevated cardiovascular risk. The indicators included waist circumference (WC), waist-to-height ratio (WHtR), weight-to-waist index (WWI), conicity index (C-Index), and combined WC&BMI measures. WC was classified as indicative of abdominal obesity (WC ≥ 88 cm) [28]. The WHtR was calculated by dividing WC (m) by height (m), with a cutoff point of WHtR > 0.50 [29,30,31]. WWI was calculated as WC (cm) divided by the square root of weight (kg) [32], with a cutoff point at the 75th percentile (WWI ≥ 11) [33,34]. The C-Index was calculated as WC (m)/[0.109 × √(weight (kg)/height (m))] [35], and the 75th percentile was also used as the cutoff (C-Index ≥ 1.27) [36,37]. Additionally, the combined WC&BMI was analyzed as a measurement of overall obesity [38], defined as the coexistence of abdominal obesity (WC ≥ 88 cm) and general obesity, according to the standard cutoff for body mass index (BMI ≥ 30 kg/m^2^) [27]. Body mass index (BMI) was calculated as weight (kg) divided by height (m) squared.

### 2.5. Main Exposures: Workday Sleep Deprivation and Weekend Catch-Up Sleep

The main exposures in this study were sleep deprivation and weekend catch-up sleep. Sleep duration was calculated based on the participants’ self-reported usual bedtimes and wake-up times over the previous month using responses to the standardized questions ‘What time do you go to sleep?’ and ‘What time do you get up?’. The participants responded in hours and minutes (hh: mm) separately for workdays and weekends (free days). Sleep duration was initially recorded in hours and minutes and subsequently converted into minutes for statistical analyses. For ease of interpretation, results are presented in decimal hours. Workday sleep deprivation was defined as a sleep duration of less than six hours per day (<6 h) on workdays [11,39]. In addition, the variation in sleep duration between workdays and weekends was assessed. Based on previous studies, ‘weekend catch-up sleep’ was calculated as the absolute difference between weekend and workday sleep durations [15,19,20]. Weekend catch-up sleep was defined as obtaining more than two additional hours of sleep (>2 h) on weekends relative to workdays [15,19,20].

### 2.6. Covariates

Data on participants’ demographic, socioeconomic, behavioral, reproductive, and occupational characteristics were collected to characterize the study population and adjust for potential confounding factors in multivariable analyses. The following demographic and socioeconomic variables were considered: age (self-reported in completed years at the time of the interview); skin color/race (self-reported and categorized as white or other—black, brown, yellow, or Indigenous); marital status (self-reported and classified as ‘without a partner’—single, separated, divorced, or widowed—or ‘with a partner’—married or in a relationship); education level (self-reported in completed years of schooling); per capita household income (calculated by summing the self-reported income all household members from the previous month, dividing by the number of residents, and expressing in multiples of the national minimum wage in 2022 [BRL 1212.00]); and head of household status (self-reported as being or not being financially responsible for the household).

Behavioral characteristics included physical activity, smoking history, alcohol consumption, and meal frequency. Physical activity was self-reported in response to the question, ‘In the past week, did you engage in any regular physical activity for leisure, sport, or exercise?’ and was categorized as ‘yes or no’. Smoking history was self-reported as nonsmoker, former smoker, or current smoker, with former and current smokers grouped together. Alcohol consumption was assessed using the question, “How often did you consume alcoholic beverages in the past year?” Responses (e.g., per day, week, month, or year) were recorded as weekly consumption and categorized as ‘no’ (no consumption or less than once per week) or ‘yes’ (consumption at least once per week). Daily meal frequency was based on the responses to the question, ‘Which meals do you usually have during the day?’ with options including breakfast, morning snack, lunch, afternoon snack, dinner, evening snack, and middle-of-the-night snack. The total number of reported meals per day was used as a dietary behavioral indicator.

Reproductive characteristics included age at menarche (self-reported in years) and parity (total number of pregnancies in a woman’s lifetime). Occupational data, including the start and end times of the participants’ work shifts, was also collected. The production sector operates in three fixed shifts: morning (from 06:00 a.m. to 02:00 p.m.), afternoon (from 02:00 p.m. to 10:00 p.m.), and night (from 10:00 p.m. to 06:00 a.m.). Administrative sector workers whose work schedules occurred during the daytime hours (from 07:00 a.m. to 07:00 p.m.) were also included. Work shifts were subsequently categorized as ‘day shift’ (from 06:00 a.m. to 10:00 p.m.) and ‘night shift’ (from 10:00 p.m. to 06:00 a.m.). An additional covariate was the average weekly sleep duration (in hours), calculated as the weighted mean based on the number of workdays and weekend days, following the formula: [(workday sleep duration × 6) + (weekend sleep duration × 1)]/7 for production workers and as [(workday sleep duration × 5) + (weekend sleep duration × 2)]/7 for administrative workers.

### 2.7. Statistical Analyses

Descriptive statistics were used to characterize the total sample and subgroups based on the outcomes and exposures. Numerical variables were summarized as means and standard deviations and/or medians and interquartile ranges (IQR), depending on the distribution of the data. Categorical variables were reported as absolute (n) and relative (%) frequencies. Group comparisons were conducted using the *t*-test for normally distributed variables and the Mann–Whitney test for non-normally distributed variables. Pearson’s chi-square test was used to assess the differences in the proportions of categorical variables. The agreement between the different abdominal adiposity indicators was assessed using the kappa (κ) statistic [40].

The association between the main exposures—workday sleep deprivation and weekend catch-up sleep—and indicators of abdominal adiposity was analyzed separately using Poisson regression with robust variance to estimate prevalence ratios (PRs) and their respective 95% confidence intervals (95% CIs). Based on conceptual models [41], four hierarchical models were considered for the inclusion of variables in the adjusted multivariate analysis: Model I (crude, unadjusted analysis); Model II, adjusted for demographic and socioeconomic variables identified as potential confounders; Model III, adjusted for Model II plus behavioral and reproductive variables; and Model IV, adjusted for Model III plus work shift (for analyses involving workday sleep deprivation as the main exposure) or by including both work shift and average weekly sleep duration (for analyses involving weekend catch-up). Potential confounders were selected a priori, and only variables associated with either the exposure or the outcome at a significance level of 0.20 were included in the multivariable analysis. Socioeconomic variables (education and income) showed no evidence of collinearity and were therefore retained in the adjusted models. Finally, potential effect modifiers of the association between sleep deprivation or weekend catch-up sleep and abdominal adiposity—including work shift (day vs. night) and work sector (administrative vs. production)—were evaluated using the Mantel–Haenszel (MH) test. No statistically significant effect modification was observed (MH test, *p* > 0.05). Likewise, sleep deprivation did not modify the association between weekend catch-up sleep and abdominal adiposity (MH test, *p* > 0.05).

All statistical analyses were performed using Stata software (version 14.0; StataCorp LP, College Station, TX, USA). Statistical significance was set at *p* < 0.05.

## 3. Results

As shown in Table 1, participants had a mean age of 34.9 ± 9.9 years; most were white (70%), without a partner (51.3%), and had 12.0 ± 2.5 years of education. The median household income was 1.2 (interquartile range [IQR]: 0.8–1.7) times the minimum wage, and 64.9% were not heads of household. Regarding health-related behaviors, most were physically inactive (71.6%), nonsmokers (76%), and reported either no alcohol consumption or consumption of less than once per week (70%). The median number of meals per day was four (IQR: 3–4). Reproductive characteristics included a mean age at menarche of 12.4 ± 1.6 years and a median parity of 1 (IQR: 0–2). Regarding occupational and sleep characteristics, most worked day shifts (78.4%) and reported a mean sleep duration of 6.6 ± 1.7 h on workdays, 8.8 ± 2.4 h on weekends, with a weekly average of 6.9 ± 1.6 h and 2.0 (IQR: 0–3.7) h of weekend catch-up sleep.

The characteristics of the participants according to the abdominal adiposity indicators are shown in Table 1. The prevalence of abdominal adiposity was 45.3% (95% CI: 40.7–49.9) based on WC, 47.6% (95% CI: 42.9–52.1) based on WHtR, 26.2% (95% CI: 22.1–30.3) for both the WWI and C-Index, and 28.7% (95% CI: 24.4–32.8) for the combined WC&BMI measure. Overall, women with abdominal adiposity were older, more often partnered, had lower education and income, and served more frequently as heads of household. They were more physically inactive, nondrinkers, had fewer meals/day, experienced earlier menarche, and had a higher parity. Additionally, they were night shift workers more often and reported shorter workdays, weekly sleep, and longer weekend catch-up sleep (Table 1). Regarding the agreement between abdominal adiposity indicators, the highest agreement was observed between WC and WHtR (91.6%; κ = 0.83), WWI and C-Index (91.1%; κ = 0.77), and WC and WC&BMI (83.3%; κ = 0.65). Moderate agreement was observed between WHtR and WC&BMI (79.8%; κ = 0.59), WC and C-Index (77.8%; κ = 0.53), WHtR and WWI (76.4%; κ = 0.52), WHtR and C-Index (76.0%; κ = 0.51), and WC and WWI (75.1%; κ = 0.48). Fair agreement was noted between C-Index and WC&BMI (74.4%; κ = 0.36) and between WWI and WC&BMI (73.6%; κ = 0.34).

Participants’ characteristics according to workday sleep deprivation and weekend catch-up sleep are presented in Table 2. Workday sleep deprivation was observed in 27.1% (95% CI: 22.9–31.2), while 43.3% (95% CI: 38.7–47.9) exhibited longer weekend catch-up sleep. Women experiencing workday sleep deprivation had lower education and income, were current or former smokers, had fewer meals/day, reported earlier menarche, were more often night shift workers, and had shorter weekends and weekly sleep but longer catch-up sleep. In contrast, those with longer weekend catch-up sleep had fewer meals/day, were night shift workers, and had shorter sleep durations on workdays, weekends, and weekly.

Table 3 presents the consistent associations between workday sleep deprivation and all abdominal adiposity indicators. After adjustment for confounders (Model IV), female fixed-shift workers experiencing sleep deprivation consistently exhibited higher probabilities of abdominal adiposity compared to those without sleep deprivation across all indicators: PR = 1.37 (95% CI: 1.10–1.69) for WC, 1.25 (95% CI: 1.02–1.53) for WHtR, 1.48 (95% CI: 1.07–2.04) for WWI, 1.43 (95% CI: 1.03–1.99) for C-Index, and 1.59 (95% CI: 1.17–2.16) for WC&BMI.

Table 4 shows the association between weekend catch-up sleep and several indicators of abdominal adiposity. Overall, no consistent significant associations were observed between these indicators. After adjusting for demographic, socioeconomic, behavioral, and reproductive confounders (Model III), significant associations were observed for WC, WHtR and WC&BMI. However, after full adjustment for potential confounders, including work shifts and weekly average sleep duration (Model IV), significant associations remained only for WHtR and WC&BMI. Specifically, female fixed-shift workers with longer weekend catch-up sleep had a higher probability of abdominal adiposity compared to those without catch-up sleep: PR = 1.24 (95% CI: 1.03–1.49) for WHtR and 1.40 (95% CI: 1.05–1.87) for WC&BMI. A borderline association was observed for WC after adjustment (PR = 1.20; 95% CI: 0.99–1.45).

## 4. Discussion

This study examined the relationships of workday sleep deprivation and weekend catch-up sleep with abdominal adiposity indicators in female fixed-shift workers. The findings revealed that workday sleep deprivation was consistently associated with a higher occurrence of abdominal adiposity across all indicators explored. In contrast, weekend catch-up sleep showed a less consistent pattern, with significant associations observed only with WHtR and WC&BMI. Notably, the prevalence of abdominal adiposity was higher in our sample of female fixed-shift workers, which is consistent with national estimates in Brazil, particularly among women [42,43].

Overall, female fixed-shift workers with sleep deprivation showed a higher probability of all abdominal adiposity indicators than those without sleep deprivation. These results align with a meta-analysis of seven prospective cohorts in general adult populations of both sexes, in which short sleep duration (mainly < 6 h) was linked to an 8% higher risk of abdominal obesity [11]. Furthermore, another meta-analysis of 21 cross-sectional studies found a significant inverse association between sleep duration and waist circumference in adult populations of both sexes, supporting the link between short sleep duration and central adiposity [44].

Several mechanisms have been proposed to explain the association between short sleep durations and increased body fat. One significant factor appears to be the alteration of endogenous hormones involved in appetite regulation and energy metabolism, which can influence body weight [45,46]. Evidence suggests that insufficient sleep is linked to elevated ghrelin, an appetite-stimulating hormone, leading to increased food consumption, weight gain, and greater fat accumulation [47,48]. Concurrently, leptin, a hormone that suppresses hunger, tends to decrease under sleep-restricted conditions [47]. These hormonal imbalances potentially intensify cravings for high-calorie, palatable foods and may also contribute to a reduction in the metabolic rate [46]. Short sleep duration may trigger adaptive metabolic and endocrine responses that increase energy intake and reduce energy expenditure, while also disrupting adipose tissue homeostasis by decreasing adiponectin levels, thereby contributing to greater fat accumulation [45]. Additionally, sleep deprivation is associated with heightened activation of the hypothalamic–pituitary–adrenal (HPA) axis, resulting in elevated cortisol levels, which further promotes fat storage [45,49,50].

Despite exploring multiple indicators of abdominal adiposity, our findings did not reveal any consistent association with weekend catch-up sleep. However, we observed a positive association between weekend catch-up sleep and both the WHtR and the combined indicators of WC&BMI. Female fixed-shift workers who reported more than 2 h of catch-up sleep on weekends showed a higher probability of abdominal adiposity. We hypothesized that this limited pattern of association may reflect a greater sensitivity of WHtR and WC&BMI in capturing central adiposity in this context or that these indicators could be more responsive to sleep-related metabolic changes, which are influenced by greater intra-individual variability in sleep duration between workdays and weekends. Our findings are consistent with those reported in a previous study [17], which identified that weekend catch-up sleep is significantly associated with increased risks of both general and abdominal obesity among Korean workers (KNHANES; n = 17,208; 26–64 y; 48.2% female), including a 21% higher risk of general obesity and an 18% higher risk of abdominal obesity compared with those with sufficient sleep, after adjustment for demographic, socioeconomic, and health-related covariates [17]. However, given the inconsistent findings in the literature regarding the relationship between weekend catch-up sleep and general obesity, our results align with those of a population-based study of Brazilian adults (SONAR-Brazil Survey; n = 2050; 18–65 y; 73.07% female). Indeed, this study has described that individuals with more than 2 h of weekend catch-up sleep had a 78% higher likelihood of being overweight and an average increase of 1.61 kg/m^2^ in BMI after adjustment for sex, age, chronotype, education level, physical activity, diet quality, and average weekly sleep duration [20].

The mechanisms underlying the relationship between weekend catch-up sleep and adiposity remain unclear, and it is uncertain whether these effects generally reflect sleep deprivation in general. A previous study indicated that insufficient sleep may lead to increased post-dinner energy intake, weight gain, delayed melatonin secretion, and reduced insulin sensitivity [51]. Moreover, ad libitum weekend recovery sleep fails to prevent metabolic dysregulation caused by repeated cycles of insufficient weekday sleep, highlighting its limited effectiveness in mitigating the adverse metabolic effects of chronic sleep deprivation [51]. Nevertheless, in our study, sleep deprivation did not modify the association between weekend catch-up sleep and abdominal adiposity, which may reflect effects other than sleep deprivation. Weekend catch-up sleep reflects sleep variability, encompassing the compensation or extension of weekdays; however, its inconsistent association with adiposity warrants cautious interpretation and further investigation.

This study examined a population of female fixed-shift workers with a notably high prevalence of abdominal adiposity as well as sleep deprivation or weekend catch-up sleep, particularly among night shift workers. Previous research has shown that shift workers are more likely to develop abdominal obesity than other obesity types, with permanent night workers at a higher risk than those working rotating shifts [26]. Similarly, high rates of abdominal adiposity have been reported among Brazilian female fixed or permanent shift workers, with a prevalence ranging from 30.2% [24] to 44.5% [52]. Moreover, the prevalence was significantly higher in female than in male shift workers (30.2% vs. 9.8%) [24]. Night-shift work is recognized as a contributor to sleep disorders in modern society. A previous review also suggested that night shifts reduce free time and sleep duration and negatively affect alertness, attention, and overall health in female night workers [53]. In our study, shift work was strongly associated with sleep deprivation, which has been linked to circadian rhythm disruption and alterations in the sleep–wake cycle, with sleep duration being significantly influenced by variations in shift start and end times [22,23]. Accordingly, current scientific literature on circadian misalignment and obesity risk has primarily focused on major disruptions to the circadian system, such as shift work and night shifts [54,55,56]. In addition, circadian misalignment produces sex-specific metabolic effects in shift workers, decreasing leptin and increasing ghrelin levels in women and increasing leptin and enhancing hedonic food desires in men [57].

These findings of this study may have important implications for public health policies and practices, particularly considering the high prevalence of abdominal adiposity among female fixed-shift workers. These policies should consider the fact that both sleep deprivation and extended weekend catch-up sleep may significantly contribute to this condition, although the absence of catch-up sleep could potentially lead to even greater adverse effects. Sleep is a fundamental pillar of health. A growing body of evidence indicates that both sleep duration and increased variability in sleep–wake patterns are significant predictors of adverse health outcomes [58,59]. Additionally, the National Sleep Foundation recommends 7–9 h of sleep per night for adults aged 18–64 years [60], and a consensus statement emphasizes that sleep regularity is essential for optimal health [14]. Thus, efforts are warranted to emphasize the role of optimal sleep duration and regularity in preventing abdominal adiposity, particularly in modern society, where factors such as extended work hours, shift work, and technology-driven lifestyles are increasingly competing with sleep.

The strengths of this study include data collection through standardized in-person interviews and data analyses that incorporate adjustments for a wide range of potential confounding factors, thereby enhancing the credibility and internal validity of the findings. A notable strength of the present study is the use of multiple objective measures of abdominal adiposity. Given that obesity is characterized by excess body fat, assessing it through body composition analysis and waist circumference [38,61], rather than relying solely on BMI, may yield more consistent associations and robust findings. Furthermore, abdominal adiposity indicators demonstrated moderate to high agreement, with the strongest concordance observed between WC and WHtR (κ = 0.83). Although research on this topic remains limited, a previous systematic review assessing concordance and correlation among anthropometric indices of obesity also reported strong agreement between WC and WHtR (κ = 0.71), suggesting that different indices may be used interchangeably to a moderate extent [62]. Accordingly, the use of varying measures of abdominal adiposity may have influenced the magnitude of these associations, although their direction remained consistent. Notably, WC emerged as the most representative indicator in our study population, given its simplicity of measurement and the strength of the associations observed.

Despite its strengths, it is important to acknowledge the limitations of this study that should be considered when interpreting its findings. First, given the correlational and cross-sectional nature of this observational study, causal inferences cannot be established, and the possibility of reverse causality cannot be excluded (e.g., sleep duration may reflect, rather than determine, abdominal adiposity). Another limitation is that sleep-related parameters, including sleep duration on workdays and free days, were assessed using self-reported questionnaires based on reported sleep onset and wake-up times, rather than objective measures such as actigraphy or polysomnography. Self-reported sleep data may overestimate sleep duration [63,64], potentially leading to an underestimation of the strength of the observed associations between sleep duration, weekend catch-up sleep, and abdominal adiposity in this study. Additionally, factors such as napping, bedtime rumination, and screen time, which are well-documented to delay sleep onset and shorten overall sleep duration in contemporary societies, were not evaluated. Future studies addressing these aspects and incorporating objective sleep assessments may further strengthen the evidence and provide more precise estimates of the observed associations. This study also relied on self-reported assessments of certain behavioral factors and did not include objective registration of these variables, such as the quantification of nicotine or cigarette consumption to characterize smoking history. In addition, potential confounding factors were not assessed, including objective measures of meal intake and other indicators of eating habits, which may influence sleep patterns and adiposity-related outcomes. Weekend catch-up sleep was calculated as the absolute difference between weekend and workday sleep duration, and participants whose weekend sleep duration was shorter than their workday sleep duration were not excluded from the analysis. These cases accounted for a relatively small proportion of the sample (11.5%). Although this approach is consistent with definitions used in previous studies [15,19,20], the inclusion of these participants may have attenuated the observed associations between weekend catch-up sleep and abdominal adiposity. Furthermore, the reasons and characteristics related to participant losses and refusals among eligible women could not be determined because the industrial group was responsible for interview logistics and prioritized the minimization of production disruption. Nonetheless, the company was informed of the inclusion and exclusion criteria of the study. Therefore, sample selection is not expected to have introduced bias, as participants’ inclusion was primarily determined by operational demands rather than by their health conditions. Finally, this study was conducted among a specific population of Brazilian female fixed-shift workers who may not be representative of other shift workers or broader population groups. Therefore, generalizations to other populations, such as male workers and those engaged in rotating or alternating shifts, should be made with caution. Nevertheless, these findings are significant and provide valuable insights for future research.

## 5. Conclusions

Workday sleep deprivation was consistently associated with a higher occurrence of abdominal adiposity across all indicators, even after adjusting for multiple confounders. These findings suggest that insufficient sleep during workdays may contribute to adverse metabolic outcomes in female workers with a fixed shift. The findings of this study also suggest that weekend catch-up sleep may be an important factor in abdominal adiposity.

Given that this study supports a significant association between sleep duration and abdominal adiposity, identifying modifiable sleep-related risk factors is essential to prevent or reduce abdominal adiposity in this specific population group. Therefore, further research is required to better understand the role of weekend catch-up sleep, its potential effects on metabolic health, and the factors that may moderate this relationship, which could inform targeted intervention strategies.

## Figures and Tables

**Table 1:** Overall characteristics of study participants and according to abdominal adiposity indicators among female fixed-shift workers (N = 450).

Characteristics		Waist Circumference (WC)			Waist-to-Height Ratio (WHtR)			Weight-to-Waist Index (WWI)			Conicity Index (C-Index)			WC (≥88 cm) & BMI (≥30 kg/m^2^)		
	Overall	<88 cm	≥88 cm	*p* ^a^	≤0.5	>0.5	*p* ^a^	<11 cm/√kg	≥11 cm/√kg	*p* ^a^	<1.27	≥1.27	*p* ^a^	No	Yes	*p* ^a^
Overall, n (%)	450 (100)	246 (54.7)	204 (45.3)		236 (52.4)	214 (47.6)		332 (73.8)	118 (26.2)		332 (73.8)	118 (26.2)		321 (71.3)	129 (28.7)	
Age, yrs																
Mean ± SD	34.9 ± 9.9	32.4 ± 9.9	38.0 ± 9.1	<0.001	32.0 ± 9.7	38.1 ± 9.3	<0.001	32.7 ± 9.6	41.2 ± 8.1	<0.001	33.0 ± 9.7	40.4 ± 8.5	<0.001	34.3 ± 10.3	36.6 ± 8.8	0.025
Skin color/race, n (%)																
White	315 (70.0)	172 (69.9)	143 (70.1)		167 (70.8)	148 (69.2)		227 (68.4)	88 (74.6)		226 (68.1)	89 (75.4)		225 (70.1)	90 (69.8)	
Other ^b^	135 (30.0)	74 (30.1)	61 (29.9)	0.967	69 (29.2)	66 (30.8)	0.711	105 (31.6)	30 (25.4)	0.207	106 (31.9)	29 (24.6)	0.134	96 (29.9)	39 (30.2)	0.946
Marital status, n (%)																
Without a partner	231 (51.3)	143 (58.1)	88 (43.1)		138 (58.5)	93 (43.5)		179 (53.9)	52 (44.1)		176 (53.0)	55 (46.6)		176 (54.8)	55 (42.6)	
With a partner	219 (48.7)	103 (41.9)	116 (56.9)	0.002	98 (41.5)	121 (56.5)	0.001	153 (46.1)	66 (55.9)	0.066	156 (47.0)	63 (53.4)	0.232	145 (45.2)	74 (57.4)	0.019
Education level, yrs																
Mean ± SD	12.0 ± 2.5	12.4 ± 2.6	11.6 ± 2.3	<0.001	12.5 ± 2.6	11.5 ± 2.3	<0.001	12.4 ± 2.6	10.9 ± 2.0	<0.001	12.2 ± 2.6	11.4 ± 2.2	0.002	12.1 ± 2.6	11.7 ± 2.3	0.084
Income ^c^, N = 446																
Median [IQR]	1.2[0.8–1.7]	1.2[0.9–1.9]	1.0[0.7–1.5]	0.003	1.2[0.9–1.9]	1.0[0.7–1.4]	<0.001	1.2[0.8–1.8]	1.0[0.8–1.4]	0.004	1.2[0.8–1.8]	1.0[0.8–1.5]	0.067	1.2[0.8–1.8]	1.0[0.8–1.4]	0.021
Head of household, n (%)																
No	292 (64.9)	174 (70.7)	118 (57.8)		166 (70.3)	126 (58.9)		225 (67.8)	67 (56.8)		224 (67.5)	68 (57.6)		218 (67.9)	74 (57.4)	
Yes	158 (35.1)	72 (29.3)	86 (42.2)	0.004	70 (29.7)	88 (41.1)	0.011	107 (32.2)	51 (43.2)	0.032	108 (32.5)	50 (42.4)	0.054	103 (32.1)	55 (42.6)	0.034
Physical activity, n (%)																
No	322 (71.6)	164 (66.7)	158 (77.4)		153 (64.8)	169 (79.0)		221 (66.6)	101 (85.6)		225 (67.8)	97 (82.2)		221 (68.8)	101 (78.3)	
Yes	128 (28.4)	82 (33.3)	46 (22.6)	0.012	83 (35.2)	45 (21.0)	0.001	111 (33.4)	17 (14.4)	<0.001	107 (32.2)	21 (17.8)	0.003	100 (31.1)	28 (21.7)	0.045
Smoking history, n (%)																
Non-smoker	342 (76.0)	190 (77.2)	152 (74.5)		183 (77.5)	159 (74.3)		253 (76.2)	89 (75.4)		252 (75.9)	90 (76.3)		241 (75.1)	101 (78.3)	
Smoker/former smoker	108 (24.0)	56 (22.8)	52 (25.5)	0.500	53 (22.5)	55 (25.7)	0.421	79 (23.8)	29 (24.6)	0.865	80 (24.1)	28 (23.7)	0.936	80 (24.9)	28 (21.7)	0.470
Alcohol consumption, n (%)																
No or less than once per week	315 (70.0)	166 (67.5)	149 (73.0)		155 (65.7)	160 (74.8)		219 (66.0)	96 (81.4)		221 (66.6)	94 (79.7)		222 (69.2)	93 (72.1)	
At least once per week	135 (30.0)	80 (32.5)	55 (27.0)	0.200	81 (34.3)	54 (25.2)	0.036	113 (34.0)	22 (18.6)	0.002	111 (33.4)	24 (20.3)	0.008	99 (30.8)	36 (27.9)	0.539
Number of meals per day																
Median [IQR]	4.0[3.0–4.0]	4.0[3.0–4.0]	4.0[3.0–4.0]	0.025	4.0[3.0–5.0]	4.0[3.0–4.0]	0.025	4.0[3.0–4.0]	4.0[3.0–4.0]	0.603	4.0[3.0–4.0]	4.0[3.0–4.0]	0.897	4.0[3.0–4.0]	3.0[3.0–4.0]	0.002
Age at menarche, years, N = 449																
Mean ± SD	12.4 ± 1.6	12.6 ± 1.5	12.0 ± 1.7	<0.001	12.6 ± 1.6	12.1 ± 1.6	<0.001	12.5 ± 1.6	12.0 ± 1.6	0.012	12.5 ± 1.6	12.1 ± 1.6	0.035	12.6 ± 1.5	11.9 ± 1.7	<0.001
Parity																
Median [IQR]	1.0[0.0–2.0]	1.0[0.0–2.0]	1.0[0.0–2.0]	<0.001	1.0[0.0–2.0]	1.0[1.0–2.0]	<0.001	1.0[0.0–2.0]	1.5[1.0–2.0]	<0.001	1.0[0.0–2.0]	1.0[1.0–2.0]	<0.001	1.0[0.0–2.0]	1.0[0.0–2.0]	0.018
Work shift, n (%)																
Day shift	353 (78.4)	203 (82.5)	150 (73.5)		192 (81.4)	161 (75.2)		265 (79.8)	88 (74.6)		263 (79.2)	90 (76.3)		261 (81.3)	92 (71.3)	
Night shift	97 (21.6)	43 (17.5)	54 (26.5)	0.021	44 (18.6)	53 (24.8)	0.115	67 (20.2)	30 (25.4)	0.234	69 (20.8)	28 (23.7)	0.504	60 (18.7)	37 (28.7)	0.020
Workday sleep duration, h																
Mean ± SD	6.6 ± 1.7	6.9 ± 1.6	6.2 ± 1.8	<0.001	6.9 ± 1.7	6.3 ± 1.7	<0.001	6.7 ± 1.7	6.2 ± 1.8	0.003	6.7 ± 1.7	6.3 ± 1.7	0.019	6.8 ± 1.7	6.1 ± 1.8	<0.001
Weekend sleep duration, h																
Mean ± SD	8.8 ± 2.4	8.7 ± 2.2	8.9 ± 2.6	0.551	8.7 ± 2.3	8.9 ± 2.5	0.283	8.8 ± 2.3	8.9 ± 2.8	0.629	8.8 ± 2.2	8.8 ± 2.8	0.862	8.7 ± 2.3	9.0 ± 2.7	0.174
Weekly average sleep duration, h																
Mean ± SD	6.9 ± 1.6	7.2 ± 1.5	6.7 ± 1.6	<0.001	7.2 ± 1.5	6.7 ± 1.6	0.001	7.1 ± 1.5	6.6 ± 1.6	0.005	7.1 ± 1.6	6.7 ± 1.6	0.023	7.1 ± 1.5	6.5 ± 1.6	<0.001
Catch-up sleep, h																
Median [IQR]	2.0[0.0–3.7]	1.6[0.0–3.0]	2.4[0.2–4.0]	0.004	1.6[0.0–3.0]	2.3[0.3–4.0]	0.002	1.8[0.1–3.4]	2.0[0.0–4.0]	0.247	1.8[0.1–3.6]	2.0[0.0–3.8]	0.440	1.7[0.0–3.1]	2.7[1.0–4.5]	<0.001

IQR, Interquartile Range. ^a^ *p*-value for Pearson’s Chi-Square Test for heterogeneity of proportions for categorical variables, *t*-test for mean difference, or non-parametric Mann–Whitney U test for variables presented as median and IQR. ^b^ Other skin color/race: black/brown/yellow/indigenous. ^c^ Per capita household income in number of minimum wages in 2022 (BRL = 1212.00). Sleep duration was initially recorded in hours and minutes and subsequently converted into minutes for statistical analyses. For ease of interpretation, results are presented in decimal hours.

**Table 2:** Characteristics of study participants according to workday sleep deprivation and weekend catch-up sleep among female fixed-shift workers, Southern Brazil, 2022. (N = 450).

Characteristics	Workday Sleep Deprivation (h/d)			Weekend Catch-Up Sleep (h)		
	≥6	<6	*p* ^a^	≤2	>2	*p* ^a^
Overall, n (%)	328 (72.9)	122 (27.1)		255 (56.7)	195 (43.3)	
Age, years						
Mean ± SD	34.7 ± 10.2	35.5 ± 9.3	0.449	34.6 ± 10.1	35.4 ± 9.8	0.360
Skin color/race, n (%)						
White	235 (71.7)	80 (65.6)		176 (69.0)	139 (71.3)	
Other ^b^	93 (28.3)	42 (34.4)	0.211	79 (31.0)	56 (28.7)	0.604
Marital status, n (%)						
Without a partner	167 (50.9)	64 (52.5)		139 (54.5)	92 (47.2)	
With a partner	161 (49.1)	58 (47.5)	0.771	116 (45.5)	103 (52.8)	0.123
Education level, years						
Mean ± SD	12.2 ± 2.6	11.5 ± 2.1	0.006	12.1 ± 2.7	11.9 ± 2.3	0.289
Income ^c^, N = 446						
Median [IQR]	1.2[0.8–1.8]	1.0[0.8–1.5]	0.026	1.1[0.8–1.7]	1.1[0.8–1.7]	0.857
Head of household, n (%)						
No	217 (66.2)	75 (61.5)		166 (65.1)	126 (64.6)	
Yes	111 (33.8)	47 (38.5)	0.355	89 (34.9)	69 (35.4)	0.915
Physical activity, n (%)						
No	231 (70.4)	91 (74.6)		185 (72.6)	137 (70.3)	
Yes	97 (29.6)	31 (25.4)	0.384	70 (27.4)	58 (29.7)	0.593
Smoking history, n (%)						
Non-smoker	259 (79.0)	83 (68.0)		196 (76.9)	146 (74.9)	
Smoker/former smoker	69 (21.0)	39 (32.0)	0.016	59 (23.1)	49 (25.1)	0.624
Alcohol consumption, n (%)						
No or less than once per week	229 (69.8)	86 (70.5)		181 (71.0)	134 (68.7)	
At least once per week	99 (30.2)	36 (29.5)	0.890	74 (29.0)	61 (31.3)	0.604
Number of meals per day						
Median [IQR]	4.0[3.0–4.0]	3.0[3.0–4.0]	<0.001	4.0[3.0–5.0]	4.0[3.0–4.0]	0.017
Age at menarche, years, N = 449						
Mean ± SD	12.5 ± 1.6	12.1 ± 1.7	0.048	12.4 ± 1.7	12.3 ± 1.5	0.390
Parity						
Median [IQR]	1.0[0.0–2.0]	1.0[0.0–2.0]	0.061	1.0[0.0–2.0]	1.0[0.0–2.0]	0.158
Work shift, n (%)						
Day shift	294 (89.6)	59 (48.4)		215 (84.3)	138 (70.8)	
Night shift	34 (10.4)	63 (51.6)	<0.001	40 (15.7)	57 (29.2)	0.001
Workday sleep duration, h						
Mean ± SD	7.4 ± 1.1	4.3 ± 0.9	<0.001	7.2 ± 1.6	5.8 ± 1.5	<0.001
Weekend sleep duration, h						
Mean ± SD	8.9 ± 2.1	8.4 ± 3.1	0.034	7.6 ± 2.0	10.4 ± 2.0	<0.001
Weekly average sleep duration, h						
Mean ± SD	7.7 ± 1.0	4.9 ± 0.9	<0.001	7.3 ± 1.6	6.5 ± 1.4	<0.001
Catch-up sleep, h						
Median [IQR]	1.6[0–3]	3.9[1.5–5.8]	<0.001	0.6[0.0–1.5]	4[3.0–5.7]	<0.001

IQR, Interquartile Range. ^a^ *p*-value for Pearson’s Chi-Square Test for heterogeneity of proportions for categorical variables, *t*-test for mean difference, or non-parametric Mann–Whitney U test for variables presented as median and IQR. ^b^ Other skin color/race: black/brown/yellow/indigenous. ^c^ Per capita household income in number of minimum wages in 2022 (BRL = 1212.00). Sleep duration was initially recorded in hours and minutes and subsequently converted into minutes for statistical analyses. For ease of interpretation, results are presented in decimal hours.

**Table 3:** Unadjusted and adjusted prevalence ratios (PR) and their respective 95% confidence intervals (95% CI) for the association between workday sleep deprivation and abdominal adiposity indicators among female fixed-shift workers, Southern Brazil, 2022. (N = 450).

Workday Sleep Deprivation (h/d)		Model I	Model II	Model III	Model IV
	n (%)	PR (95% CI)	PR (95% CI)	PR (95% CI)	PR (95% CI)
N = 450	WC ≥ 88 cm				
≥6 (n = 328)	130 (39.6)	1.00	1.00	1.00	1.00
<6 (n = 122)	74 (60.7)	1.53 (1.26–1.86)	1.43 (1.19–1.73)	1.32 (1.08–1.60)	1.37 (1.10–1.69)
N = 450	WHtR > 0.50				
≥6 (n = 328)	142 (43.3)	1.00	1.00	1.00	1.00
<6 (n = 122)	72 (59.0)	1.36 (1.12–1.65)	1.26 (1.06–1.51)	1.16 (0.97–1.39)	1.25 (1.02–1.53)
N = 450	WWI ≥ 11 cm/√kg				
≥6 (n = 328)	75 (22.9)	1.00	1.00	1.00	1.00
<6 (n = 122)	43 (35.3)	1.54 (1.13–2.11)	1.38 (1.04–1.85)	1.31 (0.98–1.17)	1.48 (1.07–2.04)
N = 450	C-Index ≥ 1.27				
≥6 (n = 328)	77 (23.5)	1.00	1.00	1.00	1.00
<6 (n = 122)	41 (33.6)	1.43 (1.04–1.97)	1.35 (1.00–1.82)	1.31 (0.97–1.77)	1.43 (1.03–1.99)
N = 450	WC ≥ 88 cm & BMI ≥ 30 kg/m^2^				
≥6 (n = 328)	77 (23.5)	1.00	1.00	1.00	1.00
<6 (n = 122)	52 (42.6)	1.82 (1.37–2.41)	1.72 (1.29–2.29)	1.53 (1.15–2.04)	1.59 (1.17–2.16)

WC, Waist circumference; WHtR, Waist-to-height ratio; WWI, Weight-to-waist index; C-Index, Conicity index; BMI, Body mass index. Model I: Unadjusted analysis (WC/WHtR/WWI/C-Index/WC &BMI). Model II: adjusted for age, marital status, education, income, and head of household (WC/WHtR/WWI/WC&BMI); adjusted for age, skin color, marital status, education, income, and head of household (C-Index). Model III: adjusted for Model II + physical activity, smoking history, alcohol consumption, number of meals per day, age at menarche, and parity (WC/WHtR/WWI); adjusted for Model II + physical activity, smoking history, number of meals per day, age at menarche, and parity (WC&BMI). Model IV: adjusted for Model III + work shift (WC/WHtR/WWI/C-Index/WC&BMI).

**Table 4:** Unadjusted and adjusted prevalence ratios (PR) and their respective 95% confidence intervals (95% CI) for the association between weekend catch-up sleep and abdominal adiposity indicators among female fixed-shift workers, Southern Brazil, 2022. (N = 450).

Weekend Catch-Up Sleep (h)		Model I	Model II	Model III	Model IV
	n (%)	PR (95% CI)	PR (95% CI)	PR (95% CI)	PR (95% CI)
N = 450	WC ≥ 88 cm				
≤2 (n = 255)	100 (39.2)	1.00	1.00	1.00	1.00
>2 (n = 195)	104 (53.3)	1.36 (1.11–1.66)	1.28 (1.06–1.56)	1.24 (1.03–1.51)	1.20 (0.99–1.45)
N = 450	WHtR > 0.50				
≤2 (n = 255)	105 (41.2)	1.00	1.00	1.00	1.00
>2 (n = 195)	109 (55.9)	1.36 (1.12–1.65)	1.28 (1.07–1.54)	1.25 (1.04–1.50)	1.24 (1.03–1.49)
N = 450	WWI ≥ 11				
≤2 (n = 255)	62 (24.3)	1.00	1.00	1.00	1.00
>2 (n = 195)	56 (28.7)	1.18 (0.87–1.61)	1.09 (0.82–1.46)	1.05 (0.79–1.40)	1.03 (0.77–1.38)
N = 450	C-Index ≥ 1.27				
≤2 (n = 255)	64 (25.1)	1.00	1.00	1.00	1.00
>2 (n = 195)	54 (27.7)	1.10 (0.81–1.51)	1.04 (0.77–1.40)	1.02 (0.76–1.37)	1.00 (0.74–1.34)
N = 450	WC ≥ 88 cm & BMI ≥ 30 kg/m^2^				
≤2 (n = 255)	58 (22.8)	1.00	1.00	1.00	1.00
>2 (n = 195)	71 (36.4)	1.60 (1.19–2.15)	1.52 (1.14–2.04)	1.47 (1.11–1.96)	1.40 (1.05–1.87)

WC, Waist circumference; WHtR, Waist-to-height ratio; WWI, Weight-to-waist index; C-Index, Conicity index; BMI, Body mass index. Model I: Unadjusted analysis (WC/WHtR/WWI/C-Index/WC&BMI). Model II: adjusted for age, marital status, education, income, and head of household (WC&BMI). Model III: adjusted for Model II + physical activity, smoking history, number of meals per day, age at menarche, and parity (WC); adjusted for Model II + physical activity, alcohol consumption, number of meals per day, age at menarche, and parity (WHtR/WWI/C-Index); adjusted for Model II + physical activity, number of meals per day, age at menarche, and parity (WC&BMI). Model IV: adjusted for Model III + work shift and weekly average sleep duration (WC/WHtR/WWI/C-Index/WC&BMI).

## Data Availability

The data presented in this study are available upon reasonable request to the corresponding author.

## Abbreviations

The following abbreviations are used in this manuscript:

BMI	Body mass index
WC	Waist circumference
WHtR	Waist-to-height ratio
WWI	Weight-to-waist index
C-Index	Conicity index
PR	Prevalence ratio
CI	Confidence interval
IQR	Interquartile ranges
